# Advanced Esophageal Neoplasm with Subcutaneous Metastasis

**DOI:** 10.1155/2019/9103137

**Published:** 2019-04-24

**Authors:** Rivadávio A. M. de Oliveira, Tassiara da Silva, Mariana Miyasaki Piovesana, Carlos Eduardo Stecca, George A. Lopes, Vitor Teixeira Liutti

**Affiliations:** ^1^Department of Clinical Oncology at the Hospital do Câncer de Londrina, Brazil; ^2^Department of Pathology Anatomy at the Hospital do Câncer de Londrina, Brazil

## Abstract

**Objective:**

This report is aimed at describing a rare clinical condition of advanced esophageal cancer with subcutaneous metastasis.

**Case Report:**

The present case refers to a patient diagnosed with stage IV esophageal squamous cell carcinoma which started with dysphonia and cervical nodules. Soon after that, the patient developed dysphagia and subcutaneous lesions on the right flank. Later in time, we documented a disease progression, with worsening of subcutaneous implants, lymph node, bone, and pulmonary metastases in addition to malignant hypercalcemia.

**Conclusion:**

This illustrates a rare presentation of an advanced esophageal neoplasm. Subcutaneous metastasis from internal malignancies is unusual, corresponding to less than 10% of cases. Its occurrence in patients with esophageal cancer is even less common with very few cases reported in literature.

## 1. Introduction

According to the American Cancer Society, there was 17,290 new cases of esophageal cancer (EC) diagnosed in 2018, representing the sixth leading cause of death by a malignant neoplasm in the world. The most common EC subtype is the squamous cell carcinoma (60%), being more frequent among African Americans [1]. It is associated with specific risk factors, including smoking, alcoholism, and a low intake of fruits and vegetables.

The major sites of EC metastasis are lymph nodes, lungs, liver, and bones. However, there was an increasing incidence of metastasis in unusual sites in recent years. It is estimated that approximately 7% of EC cases develop with cutaneous and/or muscle involvement [2].

Subcutaneous metastasis (SM) occurs in 0,6-10,4% of all patients with solid tumors [1]. Among them, breast cancer is the leading cause in women and lung cancer in men, followed by colorectal cancer with equal incidence for both sexes. Patients with SM are more susceptible to complications, such as local bleeding, infection, and refractory pain [3].

## 2. Case Report

A 41-year-old male patient with no comorbidities except history of alcoholism—consumption of distillates daily in the last twenty-five years—and smoking (60 pack-years) presented with dysphonia and right cervical lymphadenopathy in October 2017. The symptoms worsened to progressive dysphagia and odynophagia and emergence of small subcutaneous implants in the right flank. He underwent a digestive endoscopy in November 2017, which showed a 5-centimeter lesion from 20 centimeter of the upper dental arch, compatible with a well-differentiated squamous cell carcinoma on the pathological report.

**Figure 1:**
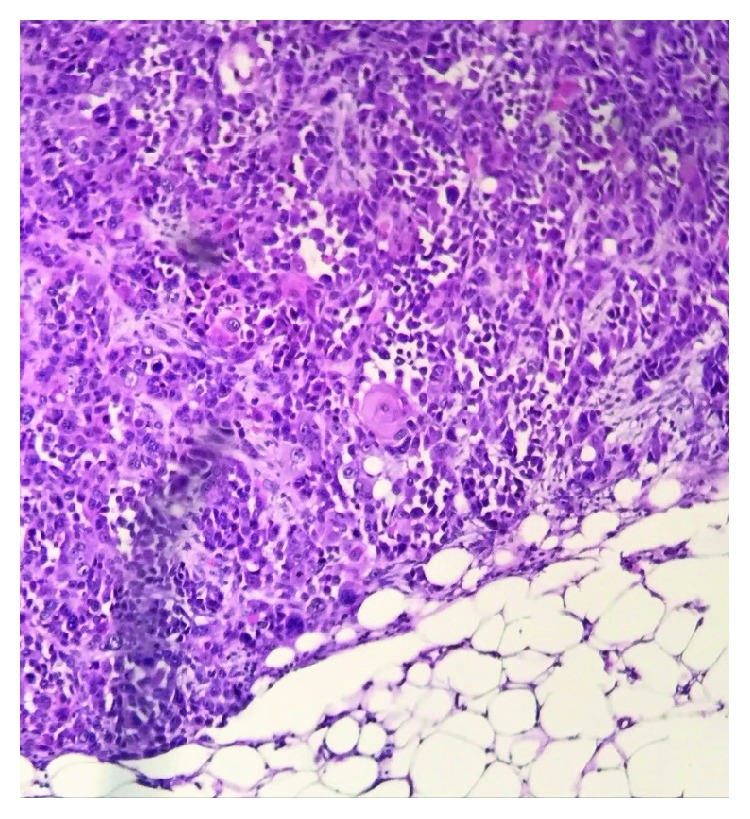
Hematoxylin and eosin stain. Anatomopathological examination of the subcutaneous nodule in the right flank. High-grade solid malignant neoplasm infiltrating the fibroadipose tissue, revealing squamous differentiation through multiple foci of keratinization.

After initial evaluation by the surgical staff, the patient was referred to exclusive treatment with clinical oncology and radiotherapy, since the tumor was not amenable to curative surgery. The staging computed tomographies demonstrated supraclavicular, mediastinal, paratracheal, infracarinal, and cervical bilateral lymphadenopathy, in addition to neoplastic implants on the right flank and right hemithorax and retroperitoneal and pleural nodules, as well as right fourth rib osteolysis. At this time, a biopsy in one of the subcutaneous implants located in the right flank was performed, confirming metastasis from esophageal squamous cell carcinoma (Figure 1) and immunohistochemistry (IHQ) was also performed, whose morphological findings were compatible with keratinizing epidermoid carcinoma infiltrating adipose tissue positive for p63/4A4 and cytokeratin cocktail.

In February 2018, the patient started his chemotherapy treatment with weekly paclitaxel and carboplatin with concomitant radiotherapy. Although metastatic, we chose this approach for a better response due to the bulky cervical disease, which engendered pain and discomfort.

**Figure 2:**
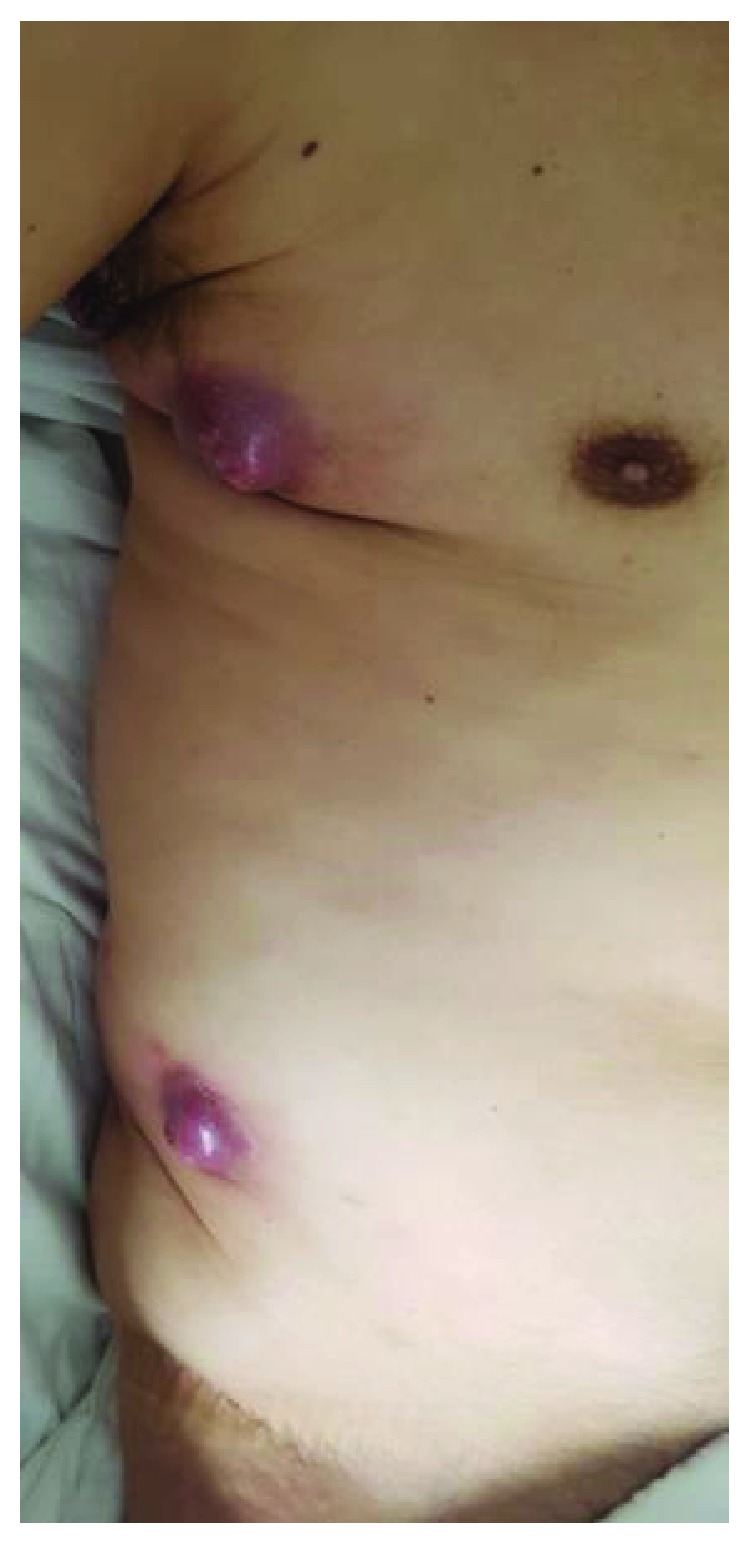
Subcutaneous metastasis on the right hemithorax and flank.

**Figure 3:**
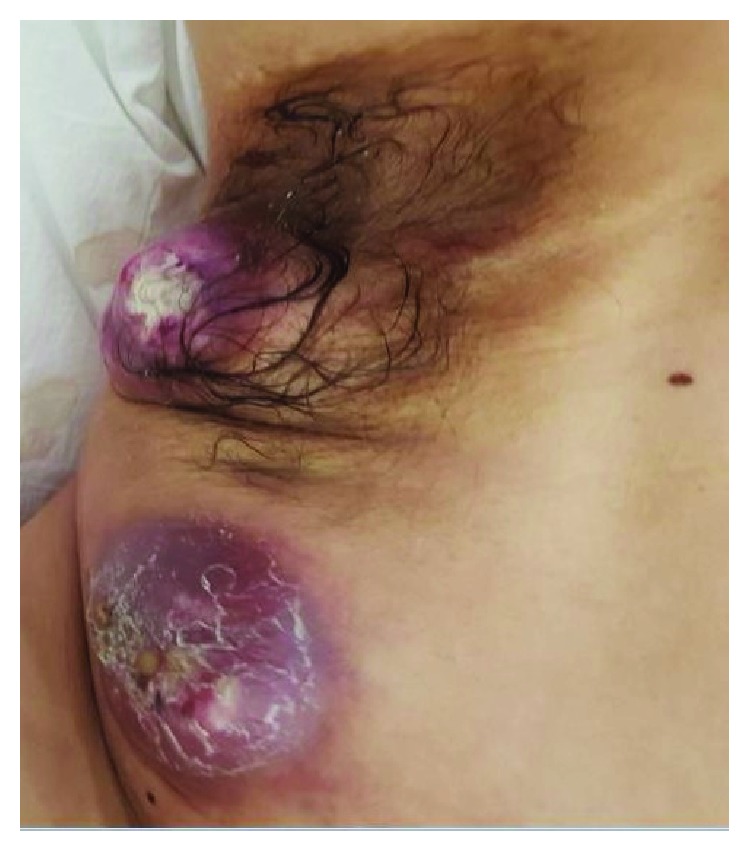
Subcutaneous metastasis in the right armpit region.

Two months after beginning his treatment, the patient developed an intense lumbar and right thigh pain, associated with walking impairment. After a medical visit, hospitalization was chosen in order to evaluate the clinical etiology. A tomography was performed, and it showed a 38 mm right paravertebral expansive lesion on the L5 level, infiltrating the psoas muscle, lesions in soft parts of the left paramedian dorsal region, worsening of the armpit and right flank subcutaneous nodules (Figures 2 and 3), and multiple lymph node, bone, and pulmonary metastases.

Since the radiologic changes would justify the symptoms reported by the patient, palliative radiotherapy was indicated. However, the patient became drowsy and confused because of malignant hypercalcemia (serum calcium of 15,5 mg/dl). Altered mental status and elevated serum calcium persisted despite hyperhydration and administration of zoledronic acid. Few days later, he developed aspiration pneumonia followed by pulmonary focus sepsis.

The septic condition, as well as hypercalcemia, proved to be refractory to the proposed treatment. Considering the advanced disease, clinical intractability, and poor-performance status, exclusive palliative care was chosen. The patient died two and a half months after beginning his treatment.

## 3. Discussion

SM are rare, since less than 1% of solid tumors cause this event [4]. Breast, lung, colorectal, and melanoma neoplasms are among the most common malignancies related to skin/subcutaneous compromise.

SM can sometimes be confused with skin infections and inadvertently treated with antibiotics, which can delay the diagnosis of a recurrent or metastatic disease [5]. SM are often painless and commonly present as papules or skin-colored, violaceous, or erythematous nodules, as presented in this case [6]. Such lesions are more frequent in men compared to women, maybe because of less health care concerns in men [7].

EC rarely presents metastasis to other sites than liver, lungs, lymph nodes, and bones [8]. SM can occur by lymphatic and hematogenic dissemination and, due to the disease aggressiveness, is associated with a worse prognosis [4]. A retrospective series of 4,020 patients with metastatic tumors found an incidence of 10% of SM, 3 of them caused by EC [9]. In the previous studies, the median survival of patients with EC at stage IV is 4 to 6 months, however, if SM is present, the life expectancy reduces to only 4 months [10, 11]. In a prior retrospective study, clinicopathological features of EC with unexpected metastasis were detailed [10]. Esophageal metastasis was disseminated toward five main anatomical sites: head and neck (42%), thoracic (17%), abdomen and pelvis (25%), extremities (9%), and multiple skin/subcutaneous and muscle metastases (7%). About two-thirds of the unexpected metastasis originated from the lower esophagus.

The present case illustrates an advanced EC of the superior thoracic esophagus with early presentation of subcutaneous metastasis and a very aggressive behavior. This is an unusual presentation since the majority of SM of EC arise from primary tumors located in the lower third of the esophagus and the average time of emergence of SM is 2,9 years after the onset of the primary tumor [4, 12].

This case report gives emphasis to a dedicated physical exam. Once SM could be a manifestation of disease in oligosymptomatic patients, it is important to suspect lesions that could be related with EC which is a way that could give an early diagnosis and better prognosis of this aggressive disease. Our case outlines the importance of biopsy and/or immunohistochemistry in the management of SM, especially in cases with atypical presentations.
